# Laser-Induced Graphene for Electrochemical Sensing of Antioxidants in Biodiesel

**DOI:** 10.1021/acsomega.4c06339

**Published:** 2024-11-19

**Authors:** Daniel R. Sevene, Tiago A. Matias, Diele A. G. Araújo, Nélio I. G. Inoque, Marcelo Nakamura, Thiago R.L.C. Paixão, Rodrigo A. A. Muñoz

**Affiliations:** † Institute of Chemistry, UFU, Federal University of Uberlândia, Uberlândia, Minas Gerais 38400-902, Brazil; ‡ Department of Chemistry, UFES, Federal University of Espírito Santo, Vitória, Espírito Santo, 29075-910, Brazil; § Institute of Chemistry, USP, University of São Paulo, São Paulo, São Paulo 05508-220, Brazil

## Abstract

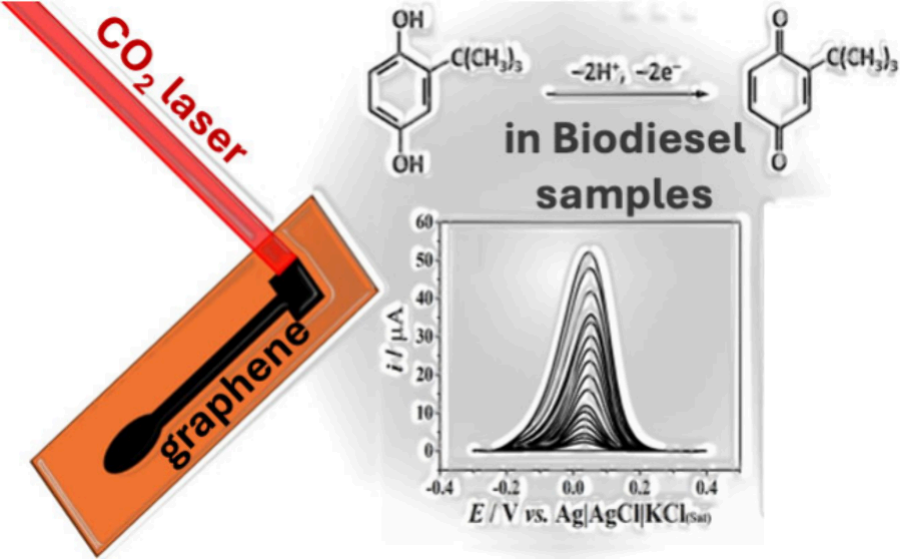

Synthetic antioxidants are often introduced to biodiesel to increase its oxidative stability, and *tert*-butyl hydroquinone (TBHQ) has been selected due to its high efficiency for this purpose. The monitoring of antioxidants in biodiesel therefore provides information on the oxidative stability of biodiesels. Herein, a laser-induced graphene (LIG) electrode is introduced as a new sensor for detecting *tert*-butyl hydroquinone (TBHQ) in biodiesel samples. An infrared CO_2_ laser was applied for LIG formation from the pyrolysis of polyimide (Kapton). Based on the voltammetric profile of a reversible redox probe, the fabrication of LIG electrodes was set using 1.0 W power and 40 mm s^–1^ speed, which presented an electroactive area of 0.26 cm^2^ (higher than the geometric area of 0.196 cm^2^). Importantly, lower engraving speed resulted in higher electroactive area, probably due to a more efficient graphene formation. Scanning-electron microscopy and Raman spectroscopy confirmed the creation of porous graphene induced by laser. The sensing platform enabled the differential-pulse voltammetric determination of TBHQ from 5 and 450 μmol L^–1^. The values of detection limit (LOD) of 2 μmol L^–1^ and RSD (relative standard deviation) of 2.5% (*n* = 10, 10 μmol L^–1^ of TBHQ) were obtained. The analysis of spiked biodiesel samples revealed recoveries from 88 to 106%. Also, the method provides a satisfactory selectivity, as it is free of interference from metallic ions (Fe^3+^, Mn^2+^, Cr^2+^, Zn^2+^, Pb^2+^, and Cu^2+^) commonly presented in the biofuel. These results show that LIG electrodes can be a new electroanalytical tool for detecting and quantifying TBHQ in biodiesel.

## 1 Introduction

Biofuels are renewable and biodegradable energy sources that are environmentally friendly and have been incorporated into diesel, enabling the use of existing engines. Due to this set of advantageous features, biodiesel has gained significant attention in recent decades as a promising alternative to conventional petroleum-based fuels.^1,2^ Crude oil reserves are limited, and fuel demand is ever-increasing. In addition, the exaggerated consumption of petroleum-derived fuels has increased the Earth’s temperature, negatively affecting life and biodiversity.^3^

The most common route to obtain biodiesel is through the catalyzed transesterification reaction of vegetable oils, recycled oils, or animal fats, forming a mixture of long-chain fatty acid esters (biodiesel itself) and glycerol.^4,5^ The obtained biodiesel does not contain sulfur and aromatic compounds; moreover, it decreases the emission of particles (HC, CO, and CO_2_) compared to diesel.^6,7^ Also, biodiesel is more vulnerable to self-oxidation than fossil fuels^8^ because it contains high quantities of unsaturated fatty acids from vegetable oil sources, like linolenic acid (approximately 8%), which are transferred to esters produced after transesterification.^7,9^

In general, the biodiesel deterioration phenomenon happens because of different causes, including oxidation by O_2(g)_ in the atmosphere by direct contact with biodiesel, thermal oxidation with as temperatures increase, microbial degradation, hydrolysis by the effect of humidity, metallic residues, which can occur separately or in combination.^10−12^ The measurement of stability against oxidation process indicates that if a biodiesel has undergone a degradation process, it changes its physical-chemical parameters and compromises its market acceptance.^13^ As a result of the low stability, biodiesel or diesel oil blends also have lower oxidative stability than pure diesel.^2,14−16^

The deterioration of biodiesel during storage can occur in two ways: (1) chemical, through reactions in the presence of air, water, light, temperature, and metals, whose reaction occurs preferentially in the unsaturated bonds of the carbon chains;^17^ (2) on account of micro-organisms, found throughout the ester, without distinction of types of chemical bonds.^18^ The Rancimat method, an accelerated oxidation method, described in European Standard (EN) 14.112, is employed to measure the oxidative stability of biodiesel in the presence of additives or not.^19,20^ These methods assess an induction period in which biofuel degradation initiates the production of volatile secondary oxidation compounds (including organic acids like formic and acetic acids), which are absorbed into a collecting solution whose conductivity is continuously monitored; a sudden increase in the conductivity marks the induction period of biodiesel oxidation.^21−23^ This process affects biodiesel properties, including acidity, flash point, cetane number, pour point, calorific value, peroxide and peroxide index, cloudiness, iodine number, and induction period. This phenomenon can lead to the formation of solid particles in biodiesel, which can deposit on the pipe walls or engine. These property changes could impact engine performance and fuel systems.^24^

**Scheme 1:**
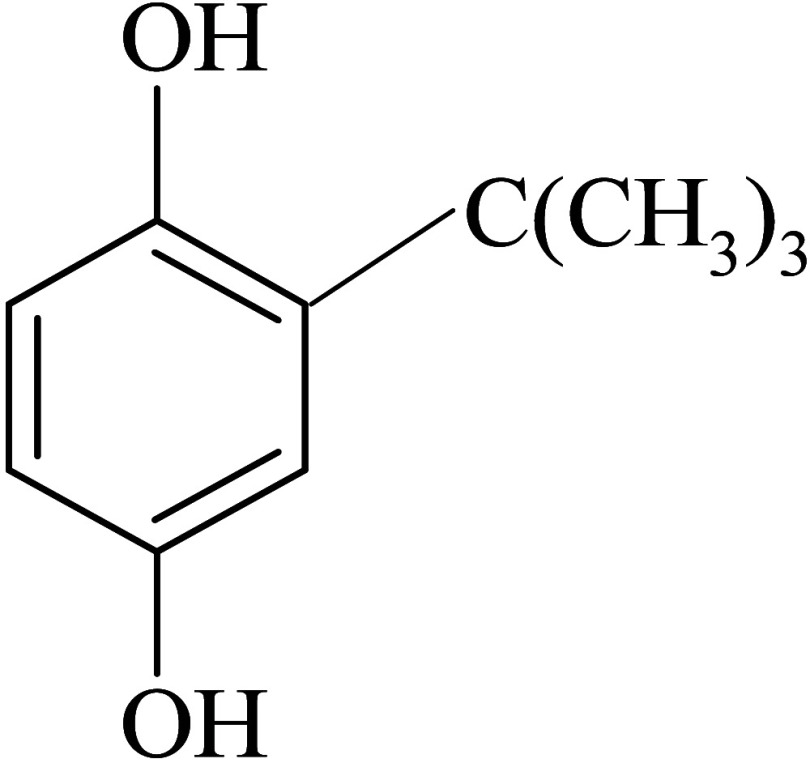
Structure of TBHQ

In view of this, oxidative stability is a decisive and determining parameter in the analysis of the final condition of biodiesel, and antioxidants are fundamental for this purpose.^25−29^ Antioxidants additives, any compound that can slow or prevent substrate oxidation at low concentrations, are used to delay the oxidative degradation of food and biofuels.^30−32^ Among many, *tert*-butyl hydroquinone (TBHQ), phenolic structure in Scheme 1, is a very popular additive that can be introduced in biodiesel during the production process to increase oxidative stability and improve market acceptance.^33−35^ The mechanism of action of TBHQ involves the donation of protons to the free radicals, avoiding the next oxidative process steps.^36−38^ Natural Gas and Biofuels (ANP), a Brazilian agency, established the limit of 12 h of induction period for the *quantum Satis* of TBHQ.^23^

The voltammetric determination of TBHQ was monitored using many electrochemical methods employing mainly carbon-based electrodes.^33,35,36,39−42^ However, the accurate determination of TBHQ in biodiesel samples using laser-induced graphene (LIG) electrodes has not yet been reported.

Research into graphene is currently leading to its successful use in applications ranging since catalysis to electronics, taking advantage of its chemical and physical properties.^43^ Graphene has been designed in three-dimensional (3D) porous structures from so many applications to offer a high surface area while maintaining its mechanical stability and high conductivity.^44,45^ The use of a commercial CO_2_ infrared laser scriber, which is an easily accessible tool that can be found in machine shops, is a an easy and scalable approach for the formation and patterning of porous 3D graphene on polyimide (PI) under ambient conditions.^46^ LIG electrodes can be manufactured in only a single step, which gives them a major advantage over conventional methods for 3D graphene synthesis.^47^ The LIG production from PI occurs through a photothermal process that starts with the localized high temperature and pressure resulting from CO_2_ laser irradiation. This results in a hierarchical porous structure on the LIG surface giving rise to a high surface area (about 340 m^2^ g^–1^), comparable to that of 3D graphene derived from wet chemistry.^46,48^ Laser parameters (power, printing speed, focus distance, etc.) have a profound effect on controlling the chemical and physical properties of LIG.^49^ In general, amplifying laser power can generate thicker LIGs while improving the conductivity. In this gratification process, sp^3^ carbon atoms form PI polymer are photothermally converted into sp^2^ carbon atoms by pulsed laser irradiation,^50^ and the resulting laser-induced graphene (LIG) exhibits high electrical conductivity. Thus, optimizing the laser parameters is important in producing high-quality and reproducible LIG electrodes.

LIG sensors allow excellent control over doping, sensitivity, short manufacturing time, low cost, and use without treatment.^51^ Their structures and composition motivated large applications, including energy storage devices, optoelectronic devices, biological and biometric devices, and chemical detection.^52−56^ Infrared-LIG-manufactured electrodes offer many advantages, such as low cost, design freedom, robustness, reproducibility, ease of operation, and portability. This work demonstrates that infrared-LIG electrodes detect antioxidants commonly added to biofuels and biodiesel. The electrochemistry response of TBHQ was investigated without any sensor surface treatment, which is a good condition for a ready-to-use sensor. The sensor was compatible with varying amounts of ethanol, which was required in the electrolyte to increase the solubility of the biodiesel prior to the voltammetric detection of TBHQ. To the best of our knowledge, the LIG electrodes have not been applied to control biodiesel quality.

## 2 Experimental Procedures

All solutions were prepared with deionized water (R ≥ 18MΩcm) obtained from a Direct-Q3 water purification system (Millipore, Bedford, MA, USA). Aqueous acids were analytical grade, used as received, and obtained from Synth (Sao Paulo, Brazil). The antioxidant *tert*-butylhydroquinone (TBHQ) (99% w/w) was purchased from Sigma-Aldrich (Steinheim, Germany). Standard TBHQ stock solutions were prepared in ethanol (99.8% v/v). Standard aqueous solutions of Cd(II), Fe(III), Cu(II), Pb(II), Zn(II), and Mn(II) (all at 1000 mg L^–1^) were purchased from Quimlab (Jacareí, Brazil). Methyl biodiesel produced from soybean oil (locally known as biodiesel) was donated by the Biodiesel Quality Control Laboratory of the Institute of Chemistry. The biodiesel was synthesized in the laboratory and according to a previous study,^57^ the biofuel did not contain any synthetic antioxidants. Analyses were performed in Britton–Robinson (BR) buffered solution (0.12 mol L^–1^; pH 6.0) with 30% Dimethylformamide (DMF).

Voltammetric measurements were conducted using a computer coupled with a Metrohm μ-AUTOLAB type III potentiostat/galvanostat (Utrecht, The Netherlands). The NOVA 2.1 software was used to control the instrument. Data were processed with the definitive software for mathematical analysis and graphing, OriginPro 8.5 (OriginLab, Northampton, MA, USA). The electrochemical cell was a 10 mL beaker, an Ag|AgCl (saturated KCl) was the reference electrode, and auxiliary electrodes were a platinum wire. The electrochemical measurements were performed at about 25 °C without removing dissolved oxygen. The electrode was used without any electrochemical treatment process. Cyclic voltammetry experiments in the presence of 1.0 mmol L^–1^ of K_3_[Fe(CN)_6_] in 0.1 mol L^–1^ KCl at scan rate of 50 mV s^–1^ and step potential of 5 mV were performed to evaluate the obtained LIG electrodes. In other experiments, the scan rate was varied from 10 to 200 mV s^–1^. Differential Pulse Voltammetry (DPV) used 0.12 mol L^–1^ Britton-Robinson (BR) as the supporting electrolyte. The optimized conditions for TBHQ determination were a modulation amplitude of 90 mV, a modulation time of 80 ms, and a step potential of 8 mV. The Raman spectroscopy measurements were performed on a confocal Raman microscope with a λ_exc_ of 633 nm from WITec.

Electrodes were prepared using a commercial laser cutter, Workspecial Laser (São Paulo, Brazil), of laser spot diameter of 150 μm and wavelength of approximately 10.6 μm. This laser cutter was used to etch and pattern conductive graphene directly onto a commercial Kapton polyimide (PI) sheet (thickness of 0.15 mm) provided by Vemar (Sorocaba, Brazil). A laser power of 900 mW with a pulse duration of 14 μs and a distance between the substrate and the laser output of 1 cm was used to etch the LIG electrodes. The laser power was measured by a power CO_2_ laser meter (CS-HLP-200, Gauge, China) with a minimal resolution of 0.1 W. The electrode design was produced using RDWorks 8.0 software, and the LIG electrode conductivity was estimated to be 1.11 ms cm^–1^. The etched area used for the working electrode was 0.196 cm^2^.

The biofuel sample was prepared by diluting it to a 1:10 biofuel/ethanol (v/v) ratio, and then, a 10 μL aliquot of the diluted biofuel was added to BR (pH 6.0) containing 30% DMF. TBHQ was determined in biodiesel by applying a standard addition procedure.

## 3 Results and Discussion

### 3.1 Fabrication and Evaluation of the Laser-Scribed Sensor

Initially, to generate the electrode, the laser power (from P7 to P11) and engraving speed (from 20 to 150 mm s^–1^) parameters were evaluated. Table S1 lists the conditions of electrode fabrication that generated LIG electrodes with the potential to be applied for electrochemical measurements. Electrodes were identified as P*x*vy, where P is the applied power, v is the engraving speed while *x* and *y* correspond to the values of power and speed applied to generate the electrode (see Table S1 for conditions of electrode fabrication). Cyclic voltammetric studies of these electrodes were carried out in 0.1 mol L^–1^ potassium chloride as the supporting electrolyte in the presence of 1 mmol L^–1^ K_3_[Fe(CN)_6_], used as the electrochemical probe. Figure S1 shows cyclic voltammograms performed on the obtained LIG electrodes. All of the fabricated electrodes responded to the redox probe. These voltammograms were analyzed based on the typical profile for a reversible diffusion-controlled redox probe involving the transference of one electron (the case of [Fe(CN)_6_]^4-/3–^), with a ratio *I*_pa_/*I*_pc_ close to 1 and anodic to cathodic peak-to-peak separation (Δ*E*) close to 59 mV at 298 K.^58,59^ For better visualization of the voltammetric data of Figure S1, Figure S2 shows a plot of *I*_pa_/*I*_pc_ and Δ*E* as a function of the scribing speed for different laser power values.

Figure S2 illustrates that LIG electrodes with Δ*E* ranging from 96 to 130 mV were produced by using varying laser power levels, which depend on the scribing speed. For example, with higher laser power (power P11), lower Δ*E* values were obtained at faster scribing speeds, while slower speeds destroyed the polyimide sheet. On the other hand, at lower power (P8), Δ*E* values showed significant variability with scribing speed, decreasing as speed increased, with the lowest Δ*E* observed at 40 and 60 mm/s. At power P9, the smallest Δ*E* was recorded at a speed of 100 mm/s, similar to P10. Thus, it is crucial to carefully control both the laser power and scribing speed for optimal voltammetric performance with this redox probe. The Δ*E* values ranged between 96 and 170 mV, which are higher than the theoretical value of 59 mV. However, LIG electrodes typically present values of Δ*E* higher than 100 mV.^60^ Higher power requires faster scribing speeds to achieve successful graphitization and prevent polyimide damage.^47,60^ Also, Figure S3 shows that the anodic (*I*_pa_) and cathodic peak currents (*I*_pc_) are directly proportional to the square root of the scan rate (ν^1/2^).^59^ The change in potential (Δ*E*_p_) was found to be approximately 75 to 105 mV as the scan rates increased from 10 to 200 mV s^–1^. This range is higher than expected (59 mV at 298 K) for a reversible electrochemical process involving a single electron. As mentioned before, Δ*E*_p_ values higher than 100 mV have been reported in the literature for LIG electrodes, so the obtained value is within the values reported in the literature.^60−62^

The electroactive area of all electrodes was fabricated, as described in Table S1 was calculated using the Randles-Sevick eq (eq 1), with [Fe(CN)_6_]^4–/3–^ as the redox probe, according to eq 1. This reversible probe was used as a reference to estimate how the power and speed influence the electroactive area:
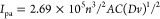
1

Where *n* = number of electrons transferred = 1, *A* = electroactive surface area of the electrode (cm^2^), *C* = concentration of the redox probe (mol L^–1^) = 0.001 mol L^–1^, *D* = diffusion coefficient of the redox probe (cm^2^/s) = 6.7 × 10^–6^ cm^2^/s, and *v* = scan rate (V/s) = 0.05 V/s.

Figures S4–S13 show the cyclic voltammograms used to calculate the electroactive area values listed in Table S2. For all laser powers applied to generate LIG electrodes (P8, P9, P10, and P11), the electroactive area decreased with the increase in scribing speed. Considering the same trend using different laser power, it can be stated that the electroactive area of LIG electrodes increases inversely with the scribing speed. The energy transferred by laser to pyrolyze polyimide depends on the laser speed and may determine the carbon forms generated, affecting the electroactive area of LIG electrodes. When a lower speed of laser engraving is applied, it can be assumed that more energy is transferred from the laser to the pyrolysis process of polyimide, generating graphene more efficiently. Pyrolysis involves the atomization of the polyimide components, leading to the release of gaseous products and the formation of a porous graphene material. This statement is confirmed by calculated electroactive area values, which are larger than the geometric area (0.196 cm^2^) in all cases (Table S2), as reported in the literature.^63^ Hence, more efficient pyrolysis results in the formation of more porous graphene, which consequently may explain the higher electroactive area of the LIG electrodes obtained at lower engraving speeds. Even though the p9v40 provided a higher electroactive area, the p8v40 was chosen due to the better voltammetric profile and best reversible parameters (Δ*E*_p_ and ratio *I*_pa_/*I*_pc_), as shown in Figure S2.

**Figure 1:**
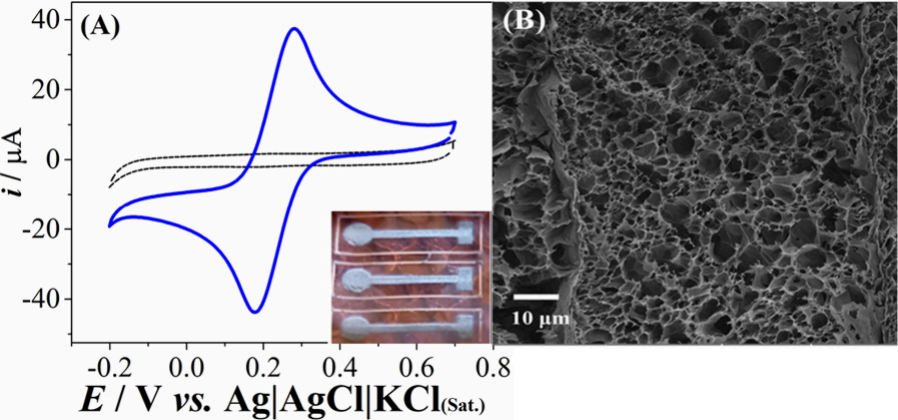
(A) Cyclic voltammogram for 1.0 mmol L^–1^ of K_3_[Fe(CN)_6_] using the LIG electrode fabricated under optimized conditions (laser power 1.0 W and engraving speed of 40 mm/s). The electrolyte sign is the dashed line. Supporting electrolyte: 0.1 mol L^–1^ potassium chloride. Voltammetric conditions: scan rate = 50 mVs^–1^ and step potential = 5 mV. The inset shows a picture of the LIG electrode (black) and polyimide polymer substrate (orange color). The circle (*r* = 2.5 mm) was used as the working electrode, which was isolated from the rest of the graphene material by applying a layer of nail polish. (B) SEM image of the LIG surface.

The selected condition for electrode preparation was 1.0 W power and 40 mm/s speed (P8v40) based on the voltammetric profile obtained for the redox probe and current intensity. A high current value and a low separation between the anodic and cathodic peaks (Δ*E* = 95.67 mV) for electrochemical probe [Fe(CN)_6_]^4–/3–^, which indicates higher electrochemical reversibility (measurement shown in Figure 1A), was obtained. Figure 1A shows that demonstrates that the LIG electrode responded consistently with the expected for a reversible diffusion-controlled redox process involving the transference of 1 with a ratio between the *I*_pa_/*I*_pc_ close to 1 (*I*_pa_ = 0.2771and *I*_pc_ = 0.18143). The SEM (scanning electron microscopy) image of the LIG electrodes (Figure 1B) showed a very regular configuration with a rough surface, conferring a high porosity, as observed in the LIG electrodes obtained by the laser-photothermal conversion of PI into graphene.^60,64^ The laser irradiation results in a localized reaction at high temperature with a break of the C=O, CO, and N=C bonds in polyimide polymer; gas are released due to the local pressure, and the graphene structures are the result of the reorganization of the remaining aromatic compounds.^46,65^ At this point, the gases released with high pressure also play a key role in the successful conversion of PI to LIG, the high pressure extinguishes the decomposition of C precursors and allows small carbon clusters to combine and form larger graphene layers.^46,66^ Moreover, the gases released can minimize the oxidation of graphitic structures in the conversion process to graphene,^67^ resulting in the formation of a porous structure, as observed by microscopy. Also, the electrodes were characterized by Raman spectroscopy, the LIG spectra (Figure S14) displayed the characteristic bands of graphene like-based materials.^64^ The D band, located at 1330 cm^–1^, is signed with the sp^2^ carbon ring breathing mode. The G band, appearing at 1580 cm^–1^, corresponds to sp^2^ in-plane carbon vibrations. The 2D band signal, originating from second-order zone-boundary phonons, arises at 2652 cm^–1^. The *I*_D_/*I*_G_ (1,20) ratio indicates the formation of highly ordered graphite with a high density of defects within graphene sheets.^68^ Also, the *I*_2D_/*I*_G_ (0.30–0.50) ratio indicates that the graphene sheets must have 4–5 layers.^68^ All these results, cyclic voltammetry, SEM, and Raman spectroscopy, indicate the conversion of polyimide to LIG after CO_2_ laser irradiation.

### 3.2 Determination of TBHQ

#### 3.2.1 Influence of pH

**Figure 2:**
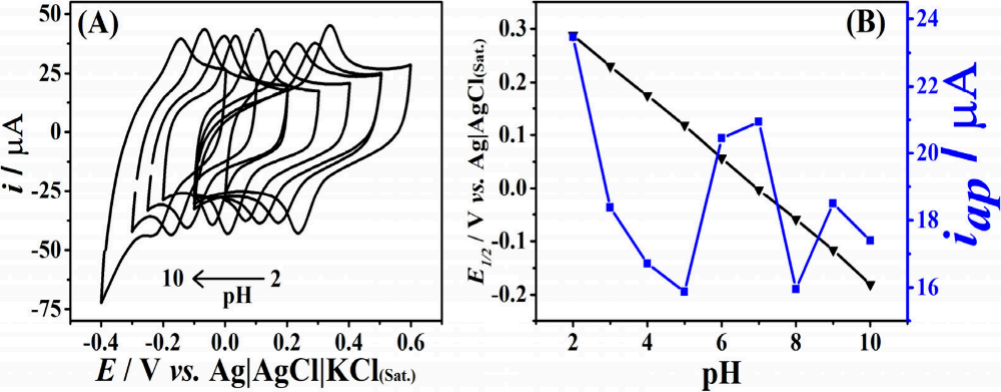
(A) Cyclic voltammograms employing LIG electrode in the presence of 50 μmol L^–1^ of [TBHQ] in 0.12 mol L^–1^ BR buffer at different pH’s. (B) Relationship between pH and half-wave potential (*E*_1/2_) and peak current (i) for the oxidation of TBHQ. CV conditions: scan rate = 50 mV s^–1^; step potential = 5 mV.

**Scheme 2:**
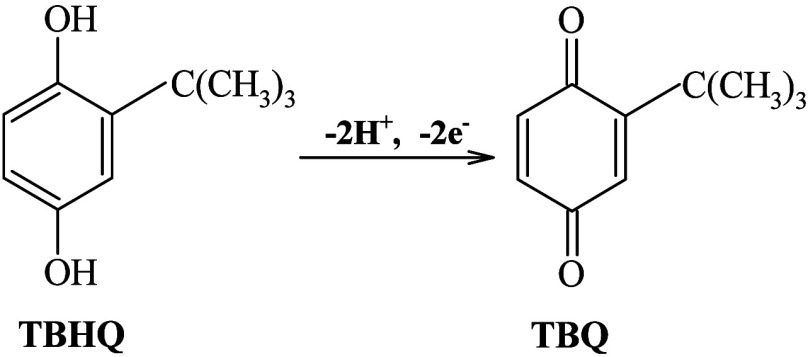
Mechanism for the 2 Protons and 2 Electrons Oxidation Process of TBHQ

The P8v40 parameter (1.0 W and 40 mm s^–1^) was selected to prepare LIG electrodes to be applied as an electrochemical sensor for TBHQ. The choice of pH of the supporting electrolyte is fundamental in voltammetric analysis for the detection of electroactive compounds.^69^ In this context, the electrochemical response of the electrode in the presence and absence of TBHQ (50 μmol L^–1^) was studied in a BR buffer solution controlling the pH from 2.0 to 10.0 by cyclic voltammetry (CV), Figure 2. Protons play an important role in electrochemical reactions and considerably affect the analyte’s peak potential and current.^69^ For the case of the laser-engraved electrode, the increase in pH resulted in the shift of the peak to a less positive potential, showing that the process was influenced by protonation.^41^ Therefore, pH 6.0 was chosen because the electrochemical response presented a higher intensity and a better definition of the cathodic and anodic peaks. A linearity of pH was observed in the of 2.0 to 10.0 range, resulting in an angular coefficient of −58.4 mVpH^–1^ (*E*_p_ = −0.0584 pH + 0.000333). For this case, the slope of 58.4 mV pH^–1^ is in agreement with the conversion of TBHQ molecule to TBQ (*tert*-butyl-p-benzoquinone), indicating that the slopes are very close to the theoretical value of 59.0 (mV pH^–1^) for a Nernstian system, which indicates that that the oxidation process involves the transfer of the same number of protons and electrons.^42,70−72^ This result is consistent with the mechanism described by Nunes Angelis,^36^ in which 2 protons and 2 electrons participate in the oxidation of TBHQ, as illustrated in Scheme 2.^40^

The electrochemical behavior of 100.0 μmol L^–1^ TBHQ in 0.12 mol L^–1^ BR buffer solution (pH 6.0) was evaluated by cyclic voltammetry (Figure S3). From Figure S3, it can be seen the TBHQ electrochemical profile, one oxidation peak at 133 mV (*E*_p,a_) and the reduction peak at 33 mV (*E*_p,c_), resulting in a separation between the anodic and cathodic peaks (Δ*E*_p_) of 100 mV. Reversibility was checked by changing the scan rate, evaluating the voltammogram profile and graph of anodic and cathodic peak current (*i*_p_) versus the square root of the scan rate (*v*^1/2^) (Figure S3C). The *I*_pa_/*I*_pc_ ratio reaches 1 (1.07), and the current intensity grows linearly as a function of the scan rate’s square root, confirming the occurrence of an electrochemical reversible system.^73^ The Figure S3D shows the logarithm plots of the current as a function of the scan rate, and it shows a linear correlation with angular coefficient values of 0.61 and 0.50 for the anodic and cathodic processes (*r* = 0.999 and 0.998, respectively). Both values are close to the theoretical value of 0.50, which suggests that the rate-determining step of the reaction is the diffusion of the TBHQ species to the electrode surface.^74^ On opposite, values close to 1.0 (theoretical value) would indicate that the rate-determining step of the reaction is the electron transfer, which was not the case of this work.

#### 3.2.2 Optimization of DPV Parameters for Determination of TBHQ

The DPV technique was selected to perform for the voltammetric determination of TBHQ. The following experiments to evaluate the DPV parameters were performed in BR buffer, pH 6.0, using the LIG electrode. The DPV parameters were optimized in the presence of 50 μmol L^–1^ of *tert*-butyl hydroquinone and 30% DMF in the solution for increasing biodiesel solubility. The parameters evaluated were modulation amplitude from 10 to 100 mV (Figure S15), modulation time from 10 to 100 mV (Figure S16), and step potential from 1 to 10 mV (Figure S17). Table 1 summarizes the DPV parameters, pH solution, studied ranges, and selected values used in all experiments. The final values were chosen considering the stability of the signal, the current response, and the resolution of the peak profile after the initial treatment.

**Table 1:** Intervals Studied and Optimized Values Selected for TBHQ Determination Using DPV

Parameters	Studied range	Optimized value
pH	2.0–10.0	6.0
Modulation Amplitude	10–100	90
Modulation Time	10–100	80
Step potential	1–10	8

**Figure 3:**
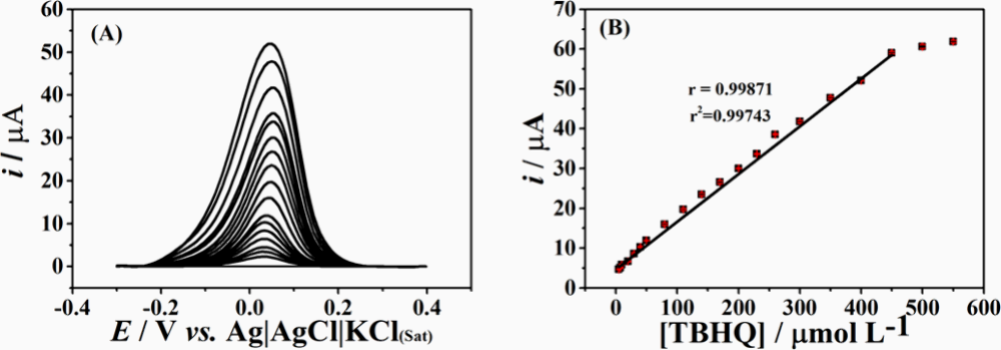
(A) Differential-pulse voltammograms with corrected baseline (*n* = 3) for increasing concentrations of TBHQ (5–550 μmol L^–1^) in BR buffer (pH = 6.0) and (B) respective calibration curve. The experiment was carried out under the optimized conditions listed in Table 1.

After optimization, an analytical curve was obtained, and the linear range for TBHQ was determined to be from 5 to 450 μmol L^–1^ (*r* = 0.999), Figure 3. The limit of detection (LOD) and limit of quantification (LOQ) were calculated according to the IUPAC (International Union of Pure and Applied Chemistry): LOD was calculated as three times the standard deviation of the intercept (σ) of the analytical curve divided by the slope of the analytical curve (s), i.e., 3σ/s, while LOQ was calculated to be 10 times the standard deviation of the intercept (σ) of the calibration curve divided by the slope of the calibration curve (s), i.e., 10σ/s. The repeatability study used the same electrode for three different TBHQ concentration levels (10, 40, and 100 μmol L^–1^). RSD values lower than 3% were obtained in all cases, indicating high precision. Table 2 lists all of the obtained analytical parameters. Thus, the proposed method based on a laser-engraved graphene sensor can be applied to determine *tert*-butyl hydroquinone in biodiesel samples.

**Table 2:** Analytical Parameters Fund from the Proposed Method for TBHQ Determination

Parameters	Optimized values
Linear range (μmol L^–1^)	5–450
Sensitivity (μA μmol^–1^ L)	0.1199 ± 0.001
Limit of detection (LOD) (μmol L^–1^)	2
Limit of quantification (LOQ) (μmol L^–1^)	7
R	0.9989
Intraelectrode (*n* = 10 for 10 μmol L^–1^)%	3
Intraelectrode (*n* = 10 for 40 μmol L^–1^)%	2
Intraelectrode (*n* = 10 for 100 μmol L^–1^)%	1

#### 3.2.3 Interference Study

**Figure 4:**
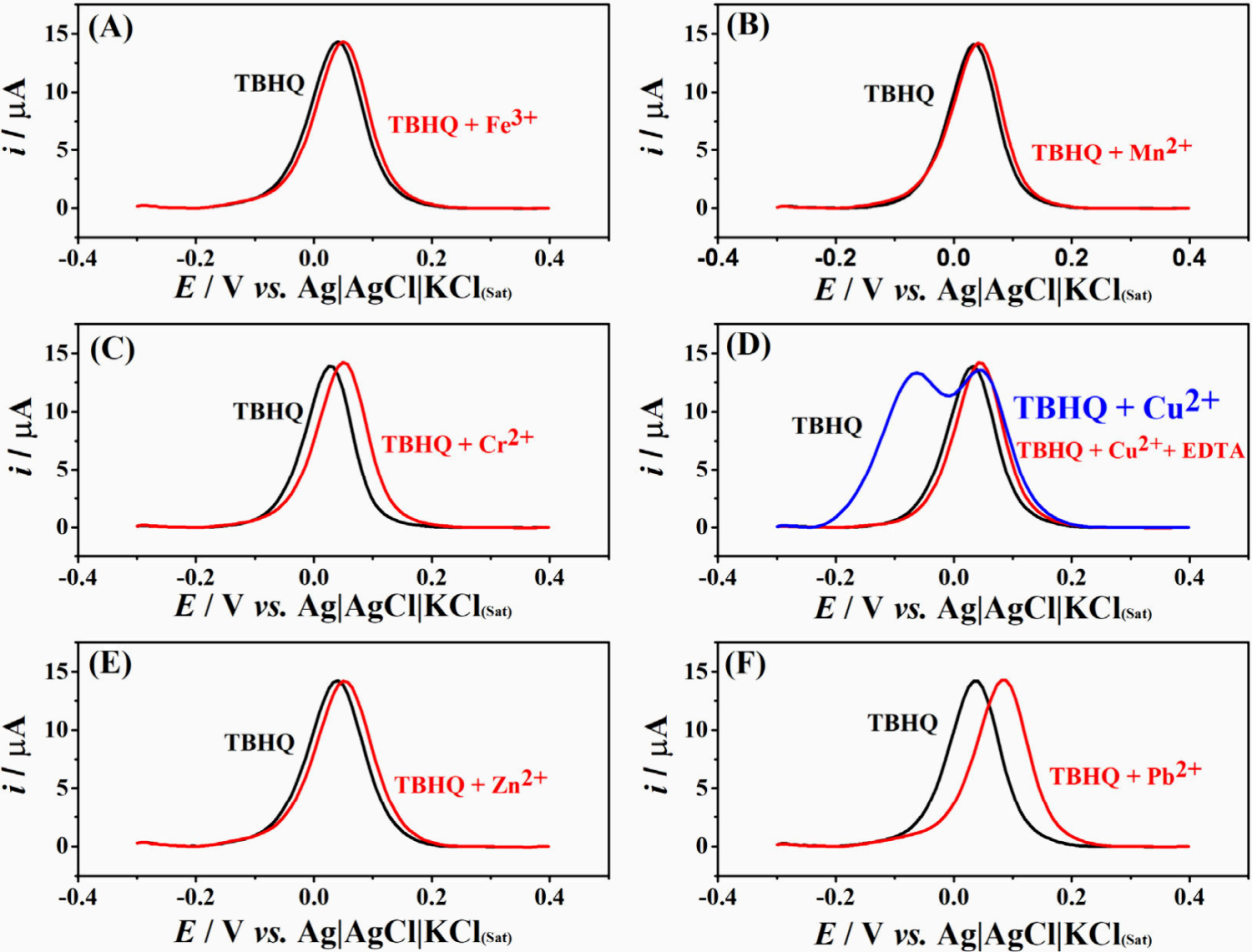
DPV (baseline corrected) obtained for 50 μmol L^–1^ of TBHQ and a mixture containing the same TBHQ contraction and 50 μg of (A) Fe^3+^, (B) Mn^2+^, (C) Cr^2+^, (D) Cu^2+^, (E) Zn^2+^, and (F) Pb^2+^, under the optimized conditions.

The selectivity of the LIG electrode was analyzed by testing its response in the presence of various metals as potential interfering species, even at low concentrations. These metals have attracted significant attention due to their negative impact on the oxidative stability of biodiesel and their role in accelerating degradation processes.^75^ Thus, engine activity and performance are hindered by the formation of insoluble salts, corrosion of components, and gum buildup, as well as by accelerated degradation in the case of biofuels.^4,76^ Additionally, various metals can be introduced into biodiesel when it comes into contact with metal components of engines or storage tanks, causing corrosion and releasing metals into the biofuel.^77−79^ Given these harmful effects, maximum limits for some contaminants, including water, sulfate, methanol, chloride, iron, and sodium, are set by regulatory agencies around the world to highlight Brazilian (ANP), European (CEN) and American (ASTM). In addition, ANP Resolution No. 842, of May 14, 2021, determined a stringent limit for the high concentration of some metal ions like Fe^3+^, Pb^2+^, Cu^2+^, Mn^2+^, and Cr^2+^ in biofuel (of 1.0 mg kg^–1^). The selectivity of the LIG electrode was studied by considering Fe^3+^, Mn^2+^, Cr^2+^, Cu^2+^, Zn^2+^, and Pb^2+^ as interferents, keeping 50 μg L^–1^ of each interfering species and 0.008 μg L^–1^ of the antioxidant TBHQ. The measurements were performed in triplicate, and the DPV measurements for these tests are shown in Figure 4. Low deviations in the signal were obtained for TBHQ in the presence of Fe^3+^, Mn^2+^, Cr^2+^, and Zn^2+^ ions. For Pb^2+^, a small shift in the potential was also registered, but this did not affect the determination of the analyte. Significantly interference with the detection of TBHQ was observed in the presence of Cu^2+^ ion; a new peak rose at around −0.1 V. To minimize copper ion interference, the samples were treated with ethylenediaminetetraacetic acid – EDTA solution at a ratio of 1:2 m/m EDTA:Cu^2+^. Ethylenediaminetetraacetic acid (EDTA) is a chelating agent and can form stable and water-soluble chelates with almost all transition metal ions over a wide pH range.^80,81^ EDTA binds to some metal ions in water, removing all the substances that harm the product formula, making it more stable and intractable than free metal ions.^82−84^ In the presence of EDTA, the interference of the Cu^2+^ ion was minimized due to the nonobservation of the signal at −0.1 V. Again, these metal species do not interfere with the detection of TBHQ under the selected conditions on the laser-engraved LIG electrode. Therefore, this sensor serves as a viable alternative to conventional methods for TBHQ determination, offering advantages such as low cost, selectivity, accuracy, and rapid fabrication without the need for additional treatment.

### 3.3 Application for Biodiesel Analysis

**Figure 5:**
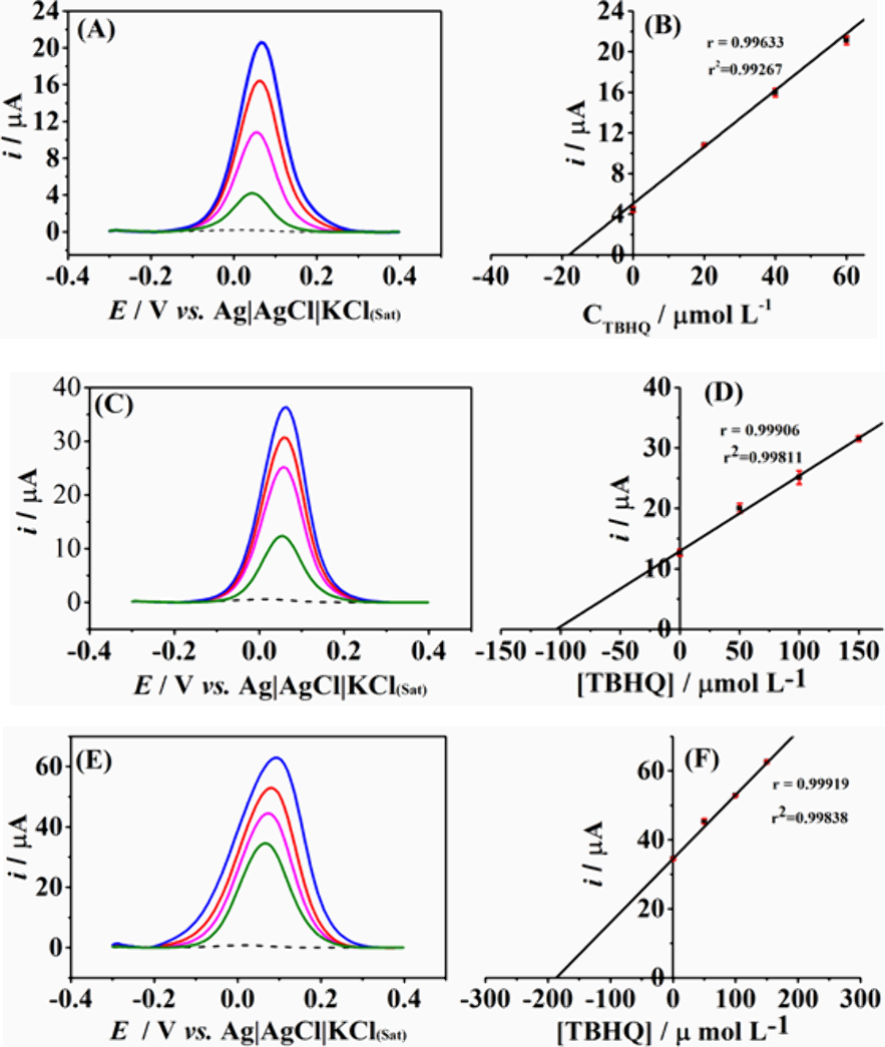
DPV records for TBHQ determination in soy biodiesel samples ((A), (C), and (E)) spiked with a standard solution resulting in final concentrations in the cell of 20 (B), 100 (D), and 200 (F) μmol L^–1^, followed by three additions of standard solutions. The respective standard addition curves are side by side in their voltammogram. The first scans show supporting electrolyte with 30% of DMF; second scan: shows spiked samples; third, fourth and fifth scans show: addition of the TBHQ standard solutions (the values can be seen in the respective curves).

After the best conditions were selected, the DPV method using the LIG electrodes was employed for TBHQ determination in soy biodiesel samples. The method’s accuracy was evaluated by recovery/addition testing. Three TBHQ concentration levels, 3.3, 16.6, and 33.0 mg L^–1^ (equivalent to concentration of 20, 100, and 200 μmol L^–1^ TBHQ in the electrochemical cell after dilution), were selected. The antioxidant concentration in the biodiesel was estimated to reach 12 h induction period measured by the Rancimat method.^34^ The curves from these analyses demonstrated satisfactory linearity (*r* > 0.99). Figure 5 presents the voltammetric measurements and the corresponding standard addition curves, while Table 3 displays the recoveries (mean values for *n* = 3) obtained from these analyses using the LIG sensor. Recovery values between 87.6% and 106% were obtained, which follows the recovery compliance criteria established by the Brazilian National Metrology, Quality and Technology Institute (INMETRO),^85^ and thus confirms acceptable accuracy at the level of concentrations studied. In addition, these results suggest that the proposed method is free of interference from the sample matrix (soy biodiesel) under the optimized conditions.

**Table 3:** Results Obtained from Recovery Experiments with TBHQ-Enriched Biodiesel Samples (*n* = 3), Showing Concentration Values Corresponding to the Amount of TBHQ in the Cell

Sample	Found (μmol L^–1^)	Fortified (μmol L^–1)^	Found ± SD (μmol L^–1)^	Recovery ± SD (%)
Biodiesel	<LOD	20	17.5 ± 0.1	87.6 ± 0.3
	<LOD	100	106 ± 8	106 ± 8
	<LOD	200	191 ± 4	96 ± 2

The procedure developed for determining TBHQ in biodiesel using the LIG electrode was compared, in terms of linear range and LOD, with other electroanalytical methods available in the literature (Table 4).

**Table 4:** Electrochemical Detection of TBHQ Employing Different Working Electrodes

aTechnique	bWorking electrode	Detection limit (μmol)	Linear range (μmol)	Ref
DPV	LIG	2	5–450	**This work**
DPV	GCE	0.57	-	(6)
SWV	Gold	1	4.76–92.40	(33)
DPV	CB/PLA	0.15	0.5–175	(35)
SWV	G/PLA	0.017	0.3–450	(39)
DPV	Platinum Ultramicro-electrode	26	29.0–361.6	(86)
LSV	SPE–MWCNT	0.34	0.5–10	(87)
SWV	HMDE	0.034	1–10	(88)
DPV	UME	212	1200–8900	(89)
MPA	GCE	5	60–600	(5)

a DPV: Differential pulse voltammetry; SWV: Square-wave voltammetry; LSV: Linear sweep voltammetry MPA: multiple-pulse amperometry.

b G/PLA: graphene-integrated polylactic acid; CB/PLA: carbon-black/polylactic acid electrode; GCE: Glassy carbon electrode; SPE–MWCNT: multiwalled carbon nanotube modified screen-printed electrodes; HMDE: Hanging mercury-drop electrode UME: platinum ultramicroelectrode.

The analyzed parameters, including working electrode type, technique, LOD, and linear range, show that the new method provides similar or better results than those already published in the literature. The proposed method presents a slightly higher LOD than some of the DPV and SWV using G/PLA, CB/PLA, GCE, Gold SPE-MWCNT, and HMDE electrodes, which obtained a LOD lower than LIG. In addition, the HDME electrode is not environmentally sound and poses a hazard to the analyst. Notably, several works have used glassy carbon, gold, and boron doped diamond electrodes, all commercial, which have a higher cost compared to the CO_2_ laser-scribed electrode used in this work. Some of these electrodes were prepared by applying more than one step, increasing the manufacturing time and affecting reproducibility.^87^ Furthermore, the CO_2_ laser printed electrode (LIG) used in this work took 60 s to manufacture and required no treatment for its application.

## 4 Conclusion

In summary, a simple, fast, and environmentally friendly protocol for producing graphene on flexible polyimide sheets for the electrochemical detection of TBHQ was introduced. The analytical performance of the LIG electrode was adequate, providing the determination and quantification of TBHQ with a linear range between 5 and 450 μmol L^–1^, and an LOD value of 2 μmol L^–1^. In addition, spiked biodiesel samples with three different concentrations were analyzed by the proposed sensor, and recovery values between 88% and 106% were obtained. Selectivity toward metallic ion interfering species was assessed, and in the critical cases (e.g., Cu^2+^) EDTA can be added to the electrolyte solutions to deplete the interference. The LIG sensor presented a similar performance compared to more expensive sensors described in the literature, which require complex and time-consuming surface treatments. Thus, these results indicate that the presented sensor manufactured from the CO_2_ laser can be used to quantify TBHQ at small concentrations and can be applied to analyze biodiesel.

## Supporting Information Available

Laser power and engraving speed evaluated to generate laser-induced graphene electrodes (Table S1); Cyclic voltammograms recorded at different parameters (Figure S1); Relationship between Δ*E* (anodic and cathodic peak-to-peak separation) (Figure S2); Voltammetric scanning rate study (Figure S3); Study of the voltammetric scan rate in the LIG recorded at P8 to P11 (Figure S4 to S13); Electroactive area of all electrodes (Table S2); Raman spectrum of LIG electrode with λ_exc_ of 633 nm (Figure S14); DPV registered for TBHQ (50 μmol L^–1^), using a 0.12 mol L^–1^ BR buffer (pH 6) as supporting electrolyte to evaluate the effect of modulation amplitude and the peak current and half peak width (Figure S15); DPV registered for TBHQ (50 μmol L^–1^), using a 0.12 mol L^–1^ BR buffer (pH 6) as supporting electrolyte to evaluate the effect of modulation time and peak current and midpeak width (Figure S16); DPV registered for TBHQ (50 μmol L^–1^), using a 0.12 mol L^–1^ BR buffer (pH 6) as supporting electrolyte to evaluate the effect of step potential and the peak current and half peak width (Figure S17). (PDF)
