# Supplementary material for: Laser-Induced Graphene for Electrochemical Sensing of Antioxidants in Biodiesel
Source: ACS Omega. 2024 Nov 19;10(1):368–77. doi: 10.1021/acsomega.4c06339 (PMC11740131; doi:10.1021/acsomega.4c06339)
Supplement: Supplementary file 1 — ao4c06339_si_001.pdf [file ao4c06339_si_001.pdf]

Supplementary material

Laser-induced graphene for electrochemical sensing of antioxidants in biodiesel

Daniel R. Sevene[1], Tiago A. Matias[2*], Diele A. G. Araújo[3], Nélio I. G. Inoque[1], Marcelo Nakamura[3], Thiago R.L.C. Paixão[3], Rodrigo A. A. Muñoz[1**].

[1] UFU, Federal University of Uberlândia, Institute of Chemistry, Uberlândia, MG, Brazil

[2]Ufes, Federal University of Espírito Santo, Department of Chemistry, Vitória, ES, Brazil

[3]USP, University of São Paulo, Institute of Chemistry, São Paulo, SP, Brazil

***Corresponding author:** * tiago.matias@ufes.br and ** munoz@ufu.br*

Supporting Information

1. Laser power and engraving speed evaluated to generate laser-induced graphene electrodes……………………….…………….……………………………………………………………………3

2. Cyclic voltammograms recorded for all electrodes…….……………………………………...………………….3

3. Relationship between ΔE (anodic and catodic peak-to-peak separation) for all electrodes…………….....….……….4

5. Voltammetric scanning rate study…………….………………………………………………………………4

7. Study of the voltammetric scan rate in the LIG recorded at P8 to P11 for Electroactive Area………………………5

4. Raman spectrum of LIG electrode……………...………………………………...…………………………………12

8. Optimization of DPV Parameters for Determination of TBHQ…………….……………………..….……………12

Table S1. Laser power and engraving speed evaluated to generate laser-induced graphene electrodes.

Electrode identification	Laser power (W)	Engraving speed (mm/s)
P7v20	0.8	20
P7v40	0.8	40
P7v60	0.8	60
P8v20	1.0	20
P8v40	1.0	40
P8v60	1.0	60
P9v40	1.4	40
P9v60	1.4	60
P9v100	1.4	100
P10v100	1.7	100
P10v150	1.7	150
P11v100	2.0	100
P11v150	2.0	150

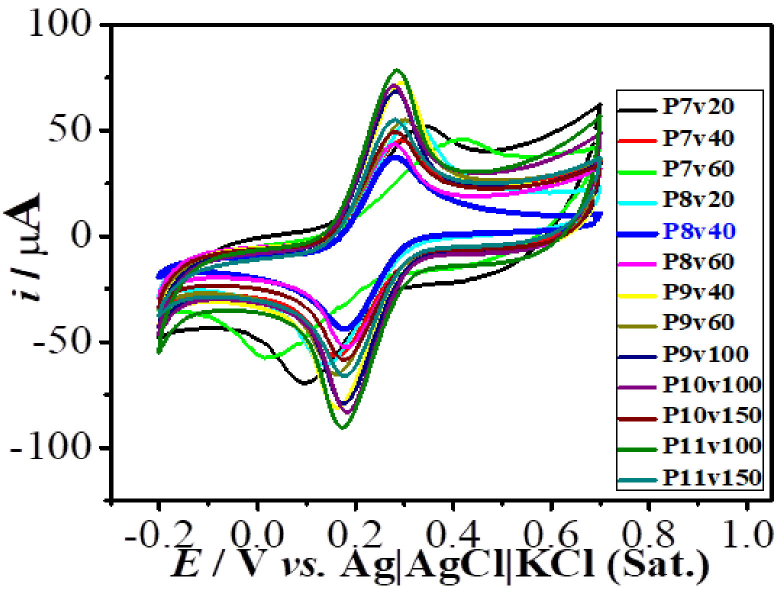

Figure S1 - Cyclic voltammograms recorded at different power (P) and scribing speed (v) parameters (P7v20, P7v40, P7v60, P8v20, P8v40, P8v60, P9v40, P9v60, P9v100, P10v100, P10v150, P11v100, P11v150,) using 1 mmol $L^{-1}$of $[Fe(CN)_6]^{4-/3-}$, using the LIG electrodes. Supporting electrolyte: 0.1 mol L^{-1} KCl.

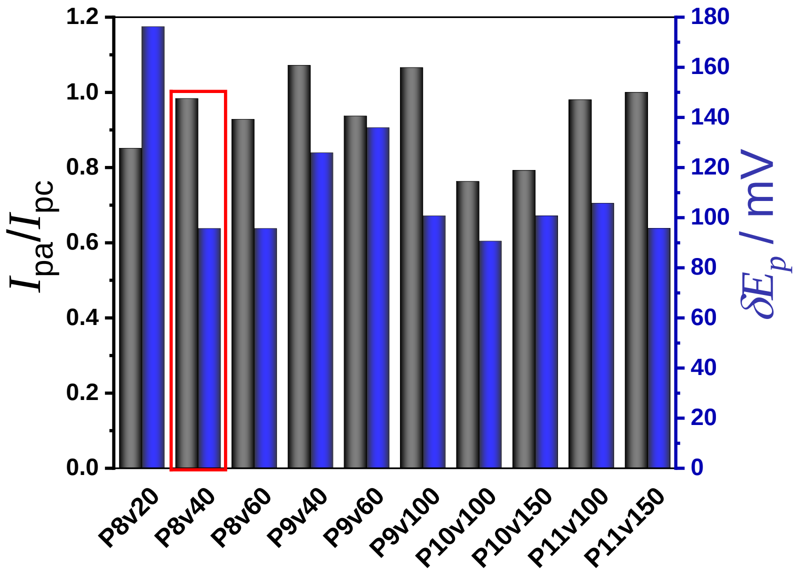

Figure S2 – Relationship between I_{pa}/I_{pc} and ΔE (anodic and cathodic peak-to-peak separation) as function of engraving speed and laser power**,** for 1 mmol L^{-1} $[Fe(CN)_6]^{4-/3-}$ using the LIG electrodes, data extracted from Figure S1. Supporting electrolyte: 0.1 mol L^{-1} KCl. The red square indicates the condition selected for the preparation of the electrodes used in this study.

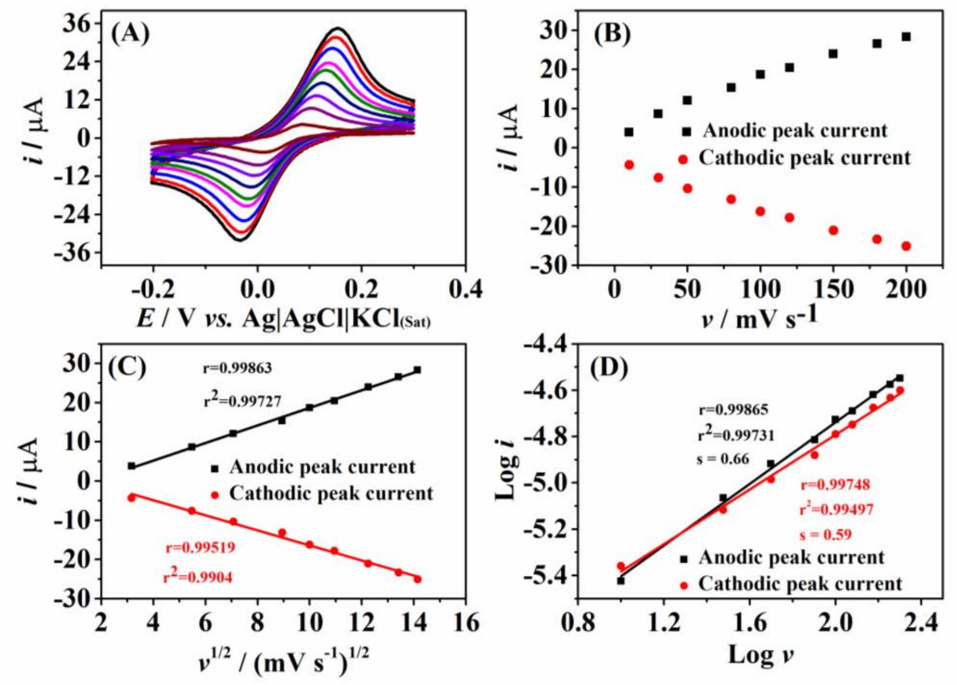

Figure S3 – Voltammetric scanning rate study: **(A)** Cyclic voltammograms obtained for the oxidation of 100 µmol L^{-1} of TBHQ in BR buffer solution 0.12 mol L^{-1} (pH = 6.0) on the LIG electrode at different scanning speeds (10 to 200 mV s^{-1}) and plots of current response as a function of: **(B)** scanning speed, **(C)** square root of scanning speed and **(D)** log i vs. log ν.

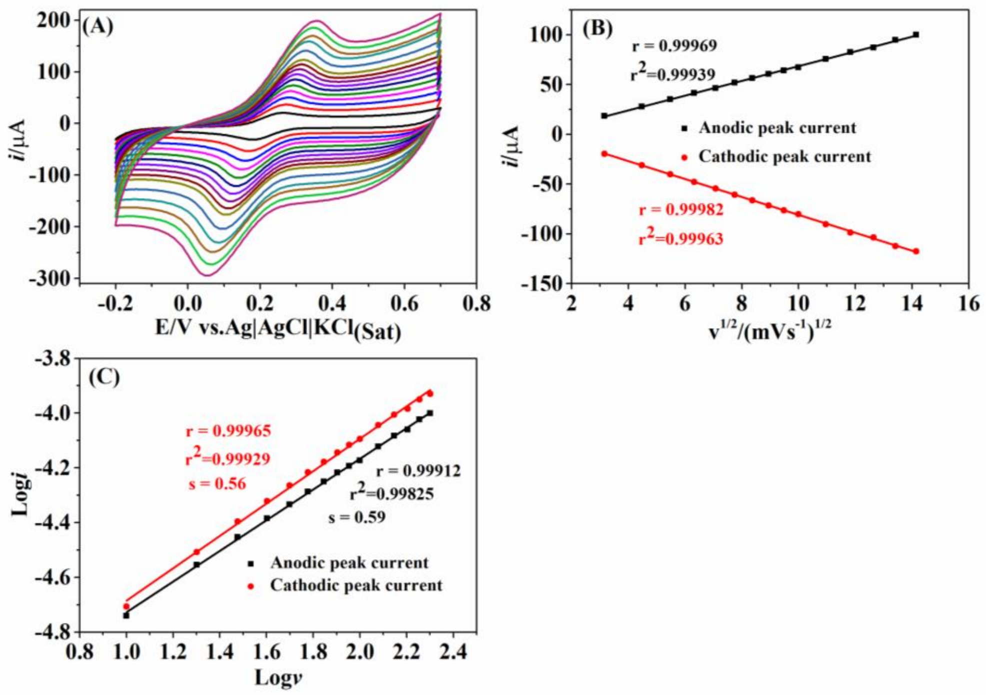

Figure S4 – Voltammetric scanning rate study at LIG recorded at P8v20: **(A)** Cyclic voltammograms obtained for the oxidation of 1 mmol L^{-1} $[Fe(CN)_6]^{4-/3-}$ on the LIG electrode at different scanning speeds (10 to 200 mV s^{-1}) and plots of current response as a function of: **(B)** square root of scanning speed and **(C)** log i vs. log ν. Supporting electrolyte: 0.1 mol L^{-1} KCl.

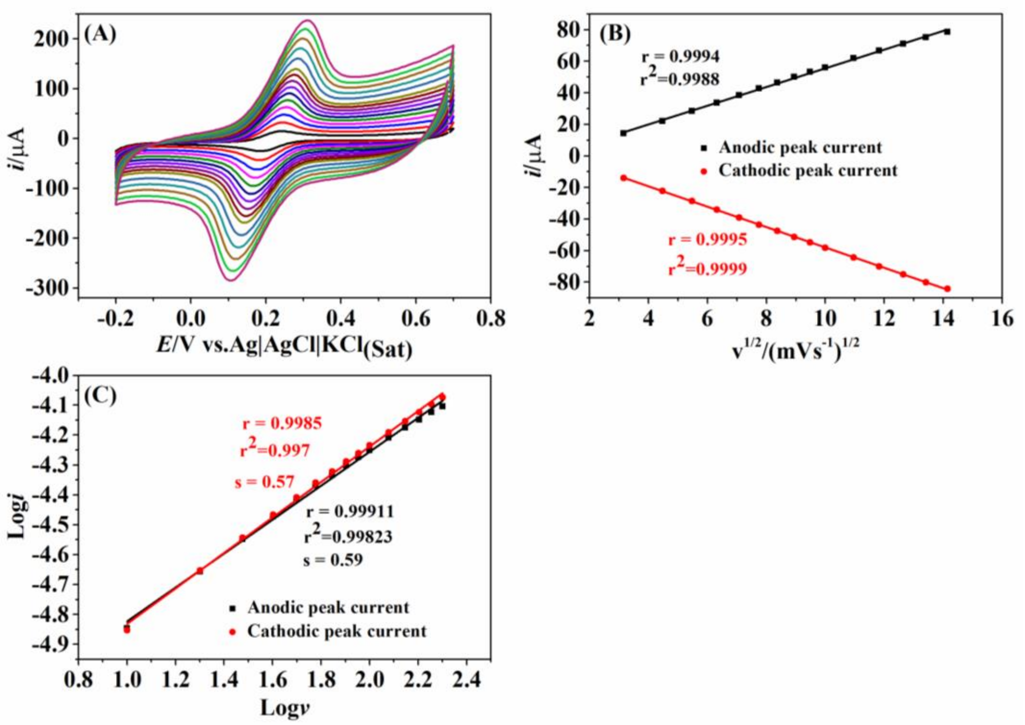

Figure S5 – Voltammetric scanning rate study at LIG recorded at P8v40: **(A)** Cyclic voltammograms obtained for the oxidation of 1 mmol L^{-1} $[Fe(CN)_6]^{4-/3-}$ on the LIG electrode at different scanning speeds (10 to 200 mV s^{-1}) and plots of current response as

a function of: **(B)** square root of scanning speed and **(C)** log i vs. log ν. Supporting electrolyte: 0.1 mol L^{-1} KCl.

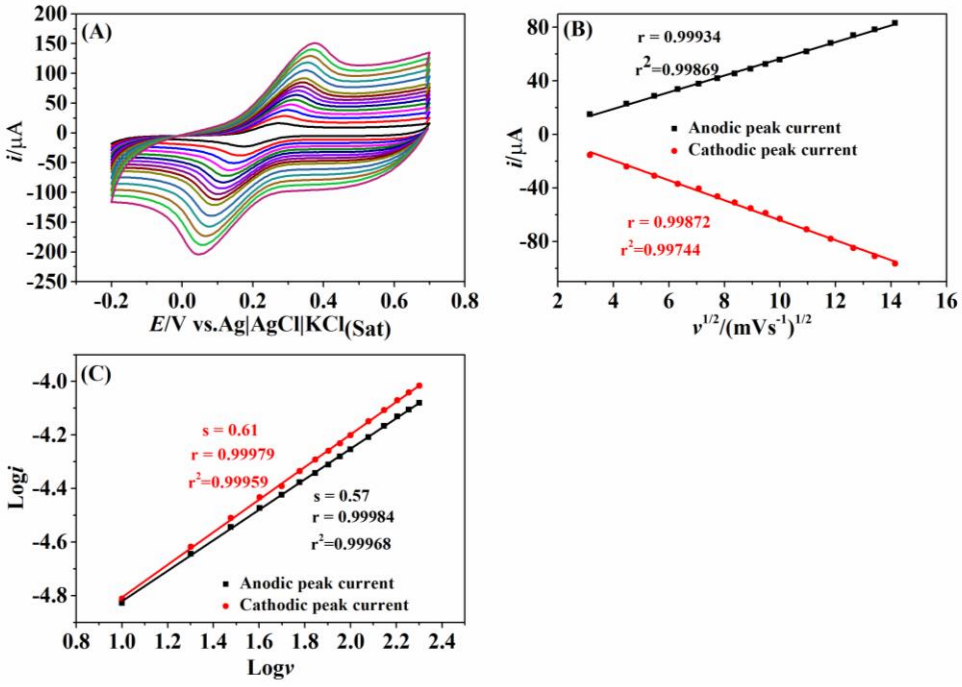

Figure S6 – Voltammetric scanning rate study at LIG recorded at P8v60: **(A)** Cyclic voltammograms obtained for the oxidation of 1 mmol L^{-1} $[Fe(CN)_6]^{4-/3-}$ on the LIG electrode at different scanning speeds (10 to 200 mV s^{-1}) and plots of current response as a function of: **(B)** square root of scanning speed and **(C)** log i vs. log ν. Supporting electrolyte: 0.1 mol L^{-1} KCl.

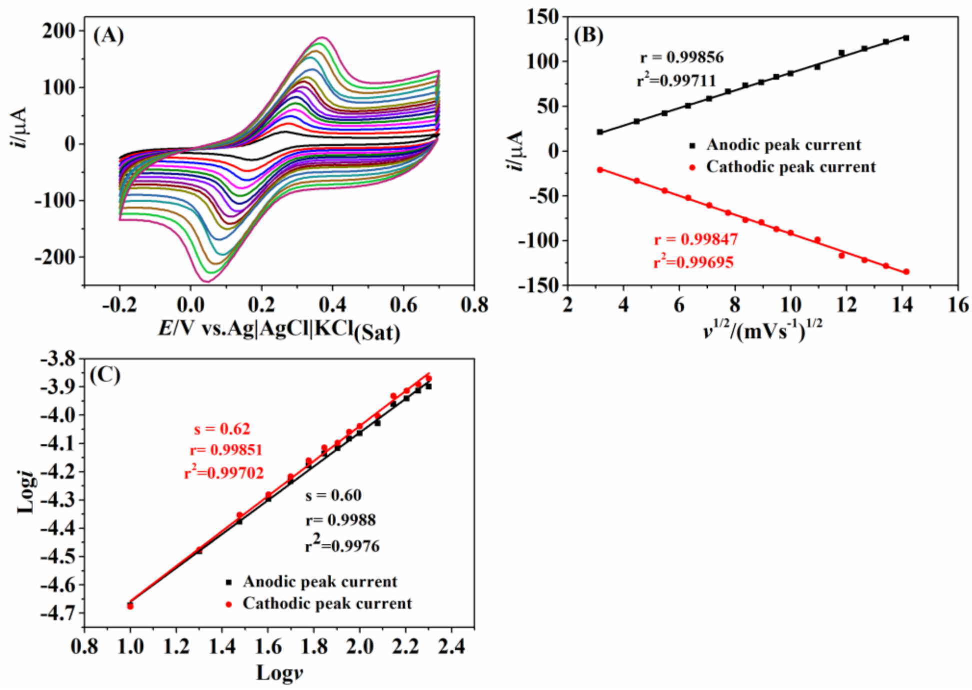

Figure S7 – Voltammetric scanning rate study at LIG recorded at P9v40: **(A)** Cyclic voltammograms obtained for the oxidation of 1 mmol L^{-1} $[Fe(CN)_6]^{4-/3-}$ on the LIG electrode at different scanning speeds (10 to 200 mV s^{-1}) and plots of current response as

a function of: **(B)** square root of scanning speed and **(C)** log i vs. log ν. Supporting electrolyte: 0.1 mol L^{-1} KCl.

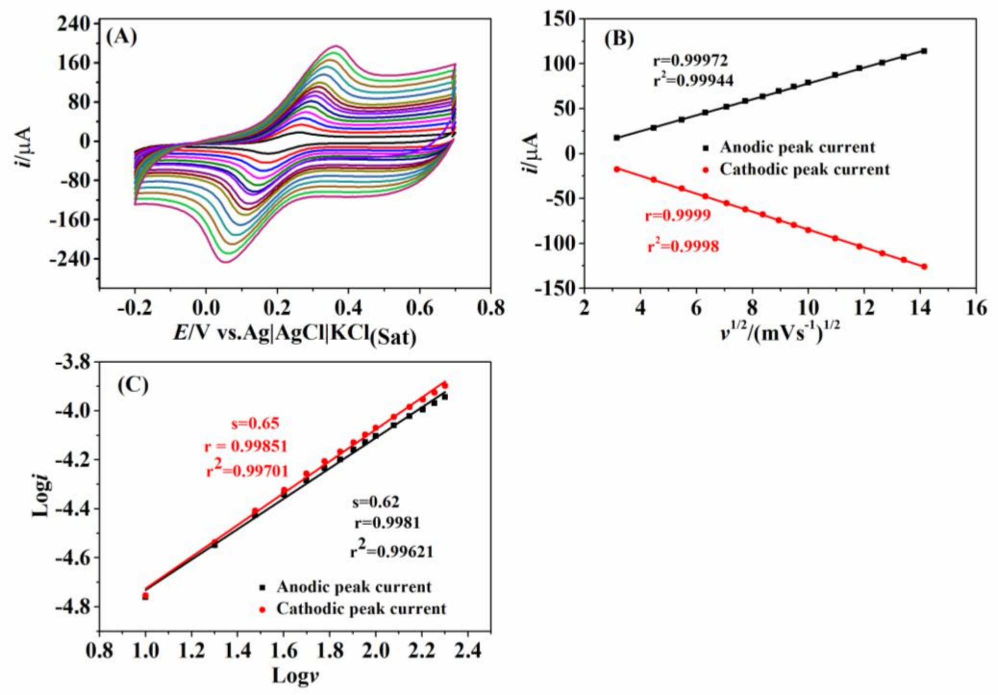

Figure S8 – Voltammetric scanning rate study at LIG recorded at P9v60: **(A)** Cyclic voltammograms obtained for the oxidation of 1 mmol L^{-1} $[Fe(CN)_6]^{4-/3-}$ on the LIG electrode at different scanning speeds (10 to 200 mV s^{-1}) and plots of current response as a function of: **(B)** square root of scanning speed and **(C)** log i vs. log ν. Supporting electrolyte: 0.1 mol L^{-1} KCl.

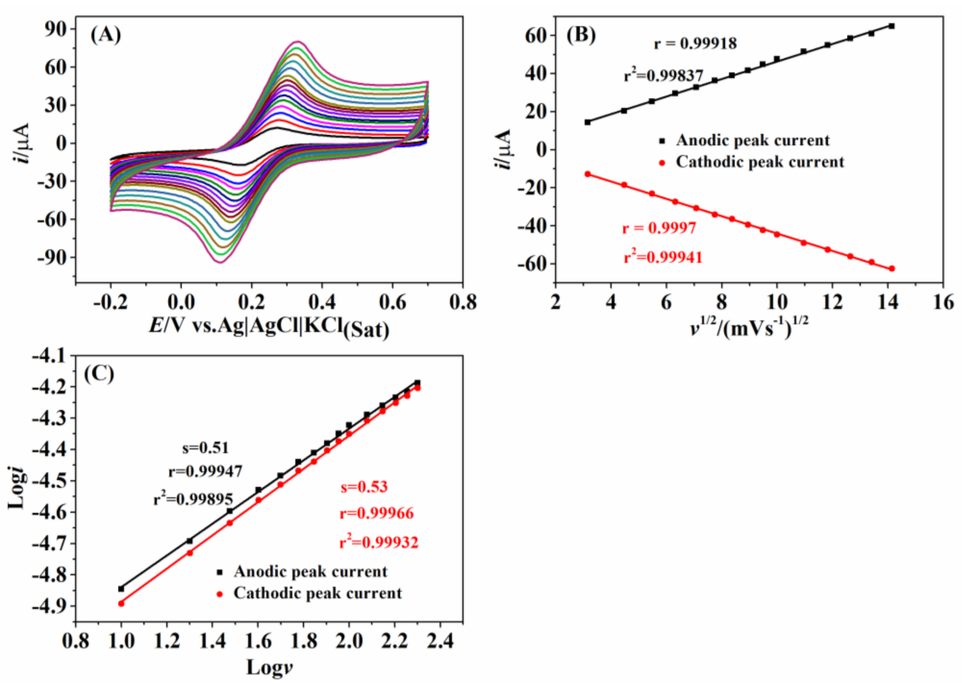

Figure S9 – Voltammetric scanning rate study at LIG recorded at P9v100: **(A)** Cyclic voltammograms obtained for the oxidation of 1 mmol L^{-1} $[Fe(CN)_6]^{4-/3-}$ on the LIG electrode at different scanning speeds (10 to 200 mV s^{-1}) and plots of current response as

a function of: **(B)** square root of scanning speed and **(C)** log i vs. log ν. Supporting electrolyte: 0.1 mol L^{-1} KCl.

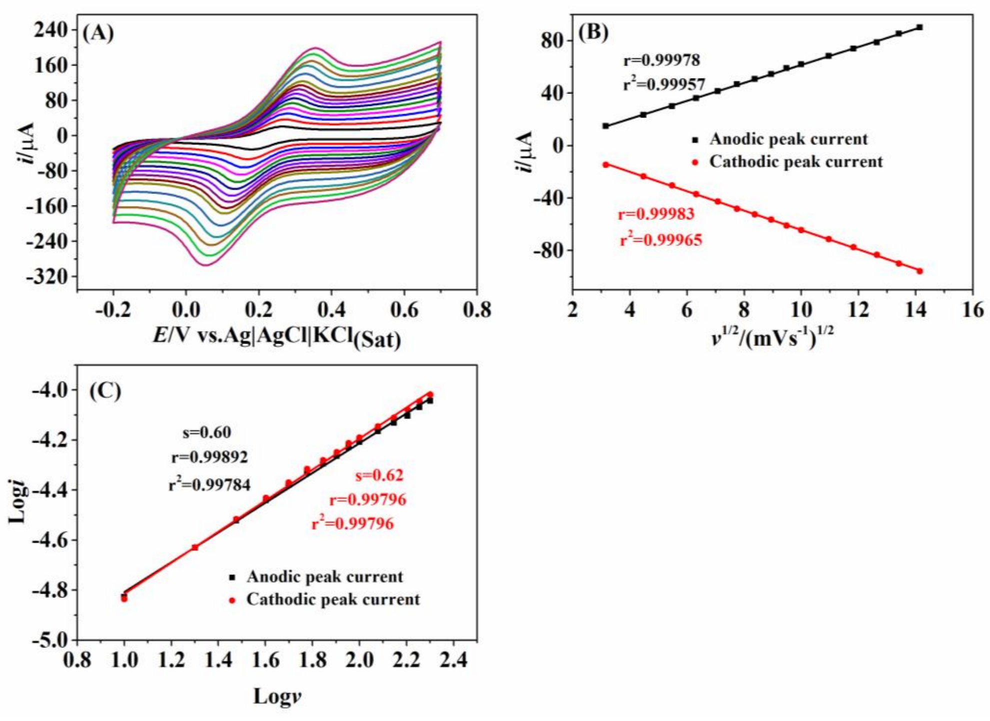

Figure S10 – Voltammetric scanning rate study at LIG recorded at P10v100: **(A)** Cyclic voltammograms obtained for the oxidation of 1 mmol L^{-1} $[Fe(CN)_6]^{4-/3-}$ on the LIG electrode at different scanning speeds (10 to 200 mV s^{-1}) and plots of current response as a function of: **(B)** square root of scanning speed and **(C)** log i vs. log ν. Supporting electrolyte: 0.1 mol L^{-1} KCl.

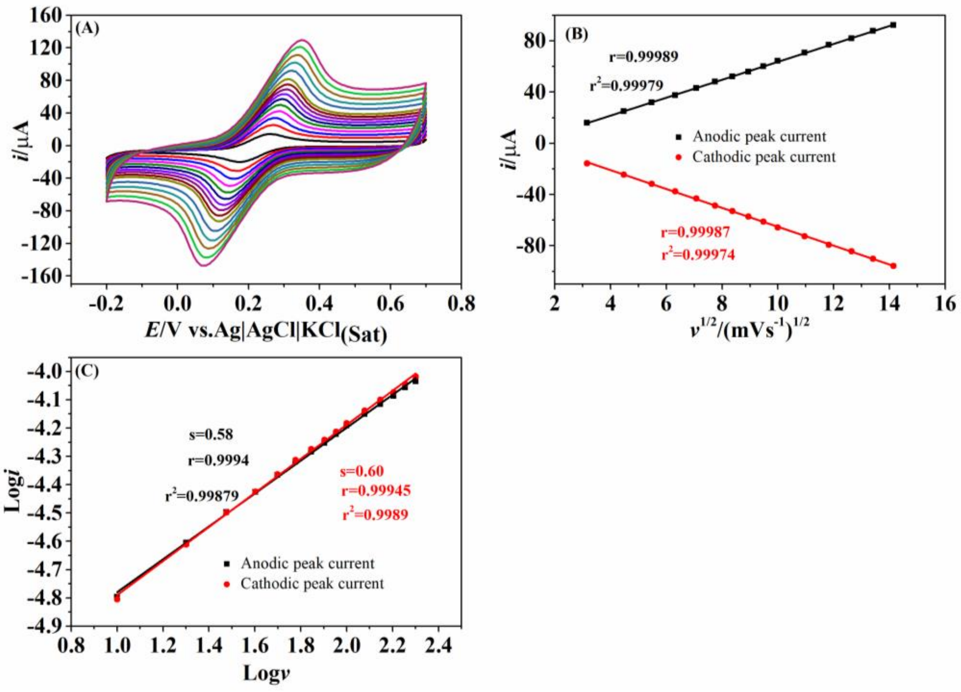

Figure S11 – Voltammetric scanning rate study at LIG recorded at P10v150: **(A)** Cyclic voltammograms obtained for the oxidation of 1 mmol L^{-1} $[Fe(CN)_6]^{4-/3}$ on the LIG

electrode at different scanning speeds (10 to 200 mV s^{-1}) and plots of current response as a function of: **(B)** square root of scanning speed and **(C)** log i vs. log ν. Supporting electrolyte: 0.1 mol L^{-1} KCl.

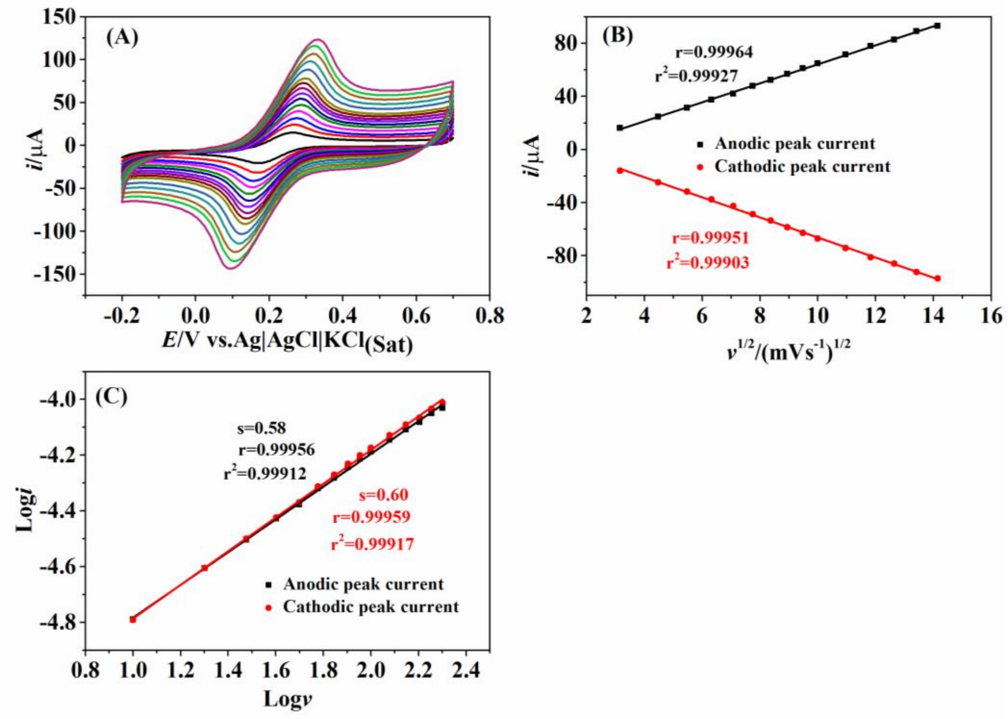

Figure S12 – Voltammetric scanning rate study at LIG recorded at P11v100: **(A)** Cyclic voltammograms obtained for the oxidation of 1 mmol L^{-1} $[Fe(CN)_6]^{4-/3-}$ on the LIG electrode at different scanning speeds (10 to 200 mV s^{-1}) and plots of current response as a function of: **(B)** square root of scanning speed and **(C)** log i vs. log ν. Supporting electrolyte: 0.1 mol L^{-1} KCl.

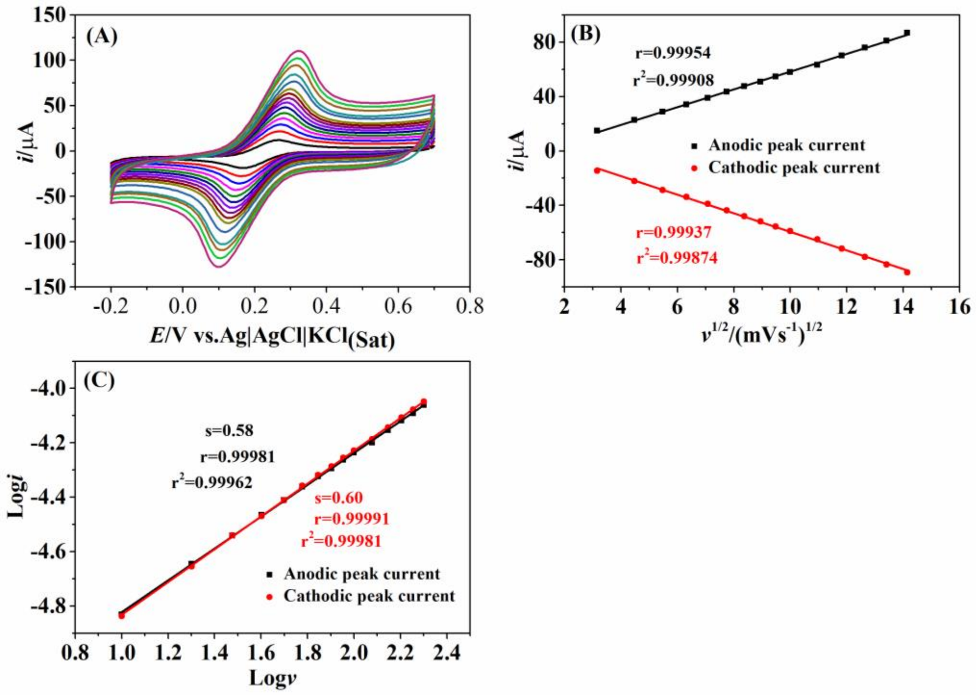

Figure S13– Voltammetric scanning rate study at LIG recorded at P11v150: **(A)** Cyclic voltammograms obtained for the oxidation of 1 mmol L^{-1} $[Fe(CN)_6]^{4-/3-}$ on the LIG electrode at different scanning speeds (10 to 200 mV s^{-1}) and plots of current response as a function of: **(B)** square root of scanning speed and **(C)** log i vs. log ν. Supporting electrolyte: 0.1 mol L^{-1} KCl.

Table S2: Electroactive area calculated by applying the Randles-Sevick equation of all electrodes, employing $[Fe(CN)_6]^{4-/3-}$ as redox probe, (data extracted form Figures S4 to S13).

Electrode	Electroactive Area (cm^2)
**P8v20**	0.37
**P8v40**	0.26
**P8v60**	0.25
**P9v40**	0.40
**P9v60**	0.36
**P9v100**	0.22
**P10v100**	0.28
**P10v150**	0.30
**P11v100**	0.29
**P11v150**	0.27

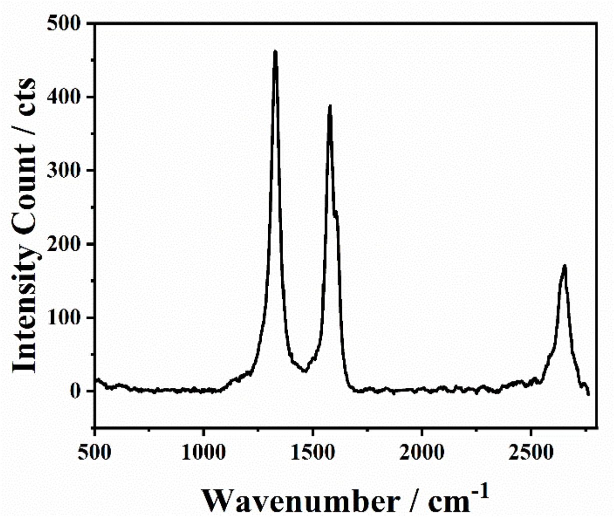

Figure S14: Raman spectrum of LIG electrode with λ_{exc} of 633 nm.

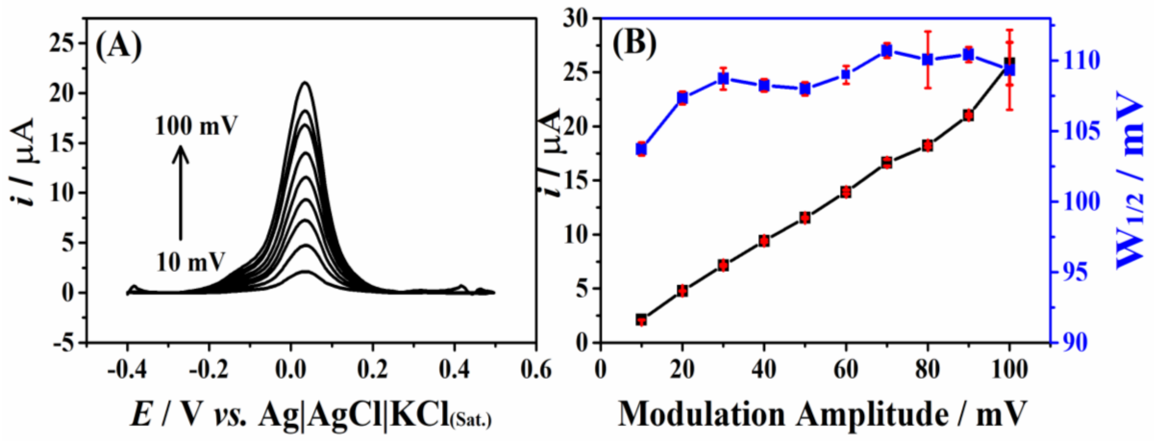

Figure S15 - (A) DPV registered for TBHQ (50 µmol L^{-1}), using a 0.12 mol L^{-1} BR buffer (pH = 6) as supporting electrolyte to evaluate the effect of modulation amplitude on **(B)** the peak current and half peak width. Other DPV conditions: modulation time: 25 ms; step potential: 5 mV.

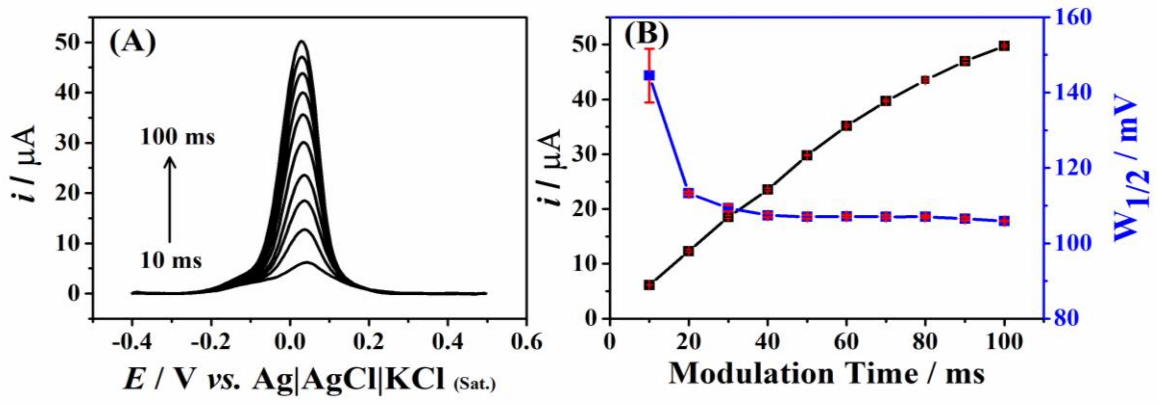

Figure S16 - (A) DPV registered for TBHQ (50 µmol L^{-1}), using a 0.12 mol L^{-1} BR buffer (pH = 6) as supporting electrolyte to evaluate the effect of modulation time on **(B)** peak current and mid-peak width. Other DPV conditions: step potential: 5 mV; modulation amplitude: 90 mV.

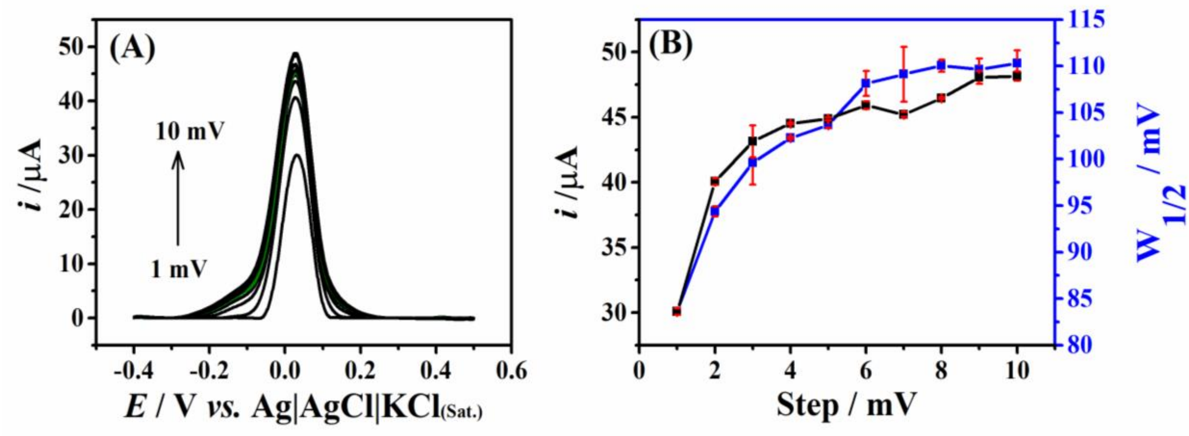

Figure S17 - (A) DPV registered for TBHQ (50 µmol L^{-1}), using a 0.12 mol L^{-1} BR buffer (pH = 6) as supporting electrolyte to evaluate the effect of step potential on **(B)** the peak

current and half peak width. Other PVD conditions: modulation amplitude: 90 mV, modulation time: 80 ms.
